# Regulating uric acid

**DOI:** 10.7554/eLife.104493

**Published:** 2024-11-11

**Authors:** Caihong Hu

**Affiliations:** 1 College of Animal Sciences, Zhejiang University Hangzhou China

**Keywords:** hyperuricemia, *Lactobacillus plantarum*, purine, nucleoside hydrolase, Mouse

## Abstract

Certain strains of a bacterium found in the gut of some animals, *Lactobacillus plantarum*, are able to counter hyperuricemia, a condition caused by high levels of uric acid in the blood.

**Related research article** Fu Y, Luo XD, Li JZ, Mo QY, Wang X, Zhao Y, Zhang YM, Luo HT, Xia DY, Ma WQ, Chen JY, Wang LH, Deng QY, Ben L, Saleemi MK, Jiang XZ, Chen J, Miao K, Lin ZP, Zhang P, Ye H, Cao QY, Zhu YW, Yang L, Tu Q, Wang WC. 2024. Host-derived *Lactobacillus plantarum* alleviates hyperuricemia by improving gut microbial community and hydrolase-mediated degradation of purine nucleosides. *eLife* **13**:e100068. doi: 10.7554/eLife.100068.

Improvements in the quality of life have led to an increase in the incidence of hyperuricemia, a medical condition that can lead to kidney stones and gout, with cases increasingly affecting younger individuals (Johnson et al., 2018; Zhang et al., 2019). Hyperuricemia – the presence of abnormally high levels of uric acid in the blood – arises from interactions between the liver, the kidneys and the gut, which has a role in removing uric acid from the body (Dalbeth et al., 2021; Niu et al., 2018; Yun et al., 2017). Studies indicate that gut microbes are crucial to uric acid metabolism, and interventions such as probiotics, prebiotics and fecal microbiota transplants can help reduce hyperuricemia by altering the gut microbiota (Cao et al., 2022a; Wang et al., 2022; Zhao et al., 2022).

It has been shown that various strains of bacteria can alleviate hyperuricemia through two mechanisms: the direct hydrolysis of uric acid, and the hydrolase-mediated degradation of nucleosides that are the precursors of uric acid in the intestine. *Limosilactobacillus fermentum* JL-3 – a strain isolated from Chinese mud water – is capable of the hydrolysis of uric acid (Wu et al., 2021), whereas various strains of *Lactobacillus*, a well-known genus of bacteria, reduce uric acid levels through the hydrolysis of nucleosides in the intestine: these strains include *L. paracasei* (X11; Cao et al., 2022b) and strains of *L. plantarum* derived from Chinese sauerkraut (DM9218-A; Li et al., 2014) and Chinese mustard (GKM3; Hsu et al., 2019).

Recent studies have revealed that gene cloning can be used to identify specific hydrolases involved in the degradation of nucleosides for *L. plantarum* and *L. aviarius* (Li et al., 2023b; Li et al., 2023a). However, the precise mechanisms underlying the hydrolysis of the nucleoside precursors of uric acid have remained unclear. Now, in eLife, Wence Wang (South China Agricultural University), Qiang Tu (Shandong University) and colleagues – including Yang Fu as first author – report the results of in vitro studies and experiments on geese and mice that shed new light on the hydrolysis of these precursors (Fu et al., 2024).

The team isolated a strain called *L. plantarum* SQ001 from geese with hyperuricemia, and a genome-wide analysis revealed the presence of four genes that code for nucleoside hydrolysis-related enzymes (iunH, yxjA, rihA, rihC). In vitro experiments revealed that one of these enzymes, iunH, effectively catalyzes the hydrolysis of nucleosides, such as inosine and guanosine, converting them to nucleobases, as evidenced by metabolomics analysis. The hydrolysis mechanism was further validated through experiments that involved knocking out the gene for iunH in *L. plantarum* SQ001, and expressing it in *E. coli*. Although nucleosides are hydrolyzed to produce nucleobases, the direct link between this process and the reduction of uric acid remains unclear, possibly due to the transport of nucleosides and nucleobases in the gut. It may be that the lower uptake of these substances reduces the synthesis and accumulation of uric acid.

The team validated the functionality of *L. plantarum* SQ001 by establishing models of hyperuricemia in both geese and mice (Figure 1), and showed that this particular strain significantly enhanced the abundance of *Lactobacillus* in the gut of the host, which alleviated the symptoms of hyperuricemia by reducing the synthesis of uric acid and increasing its excretion. The fact that hyperuricemia was alleviated in mice may help with efforts to develop new ways to treat hyperuricemia and gout in humans.

**Figure 1.**
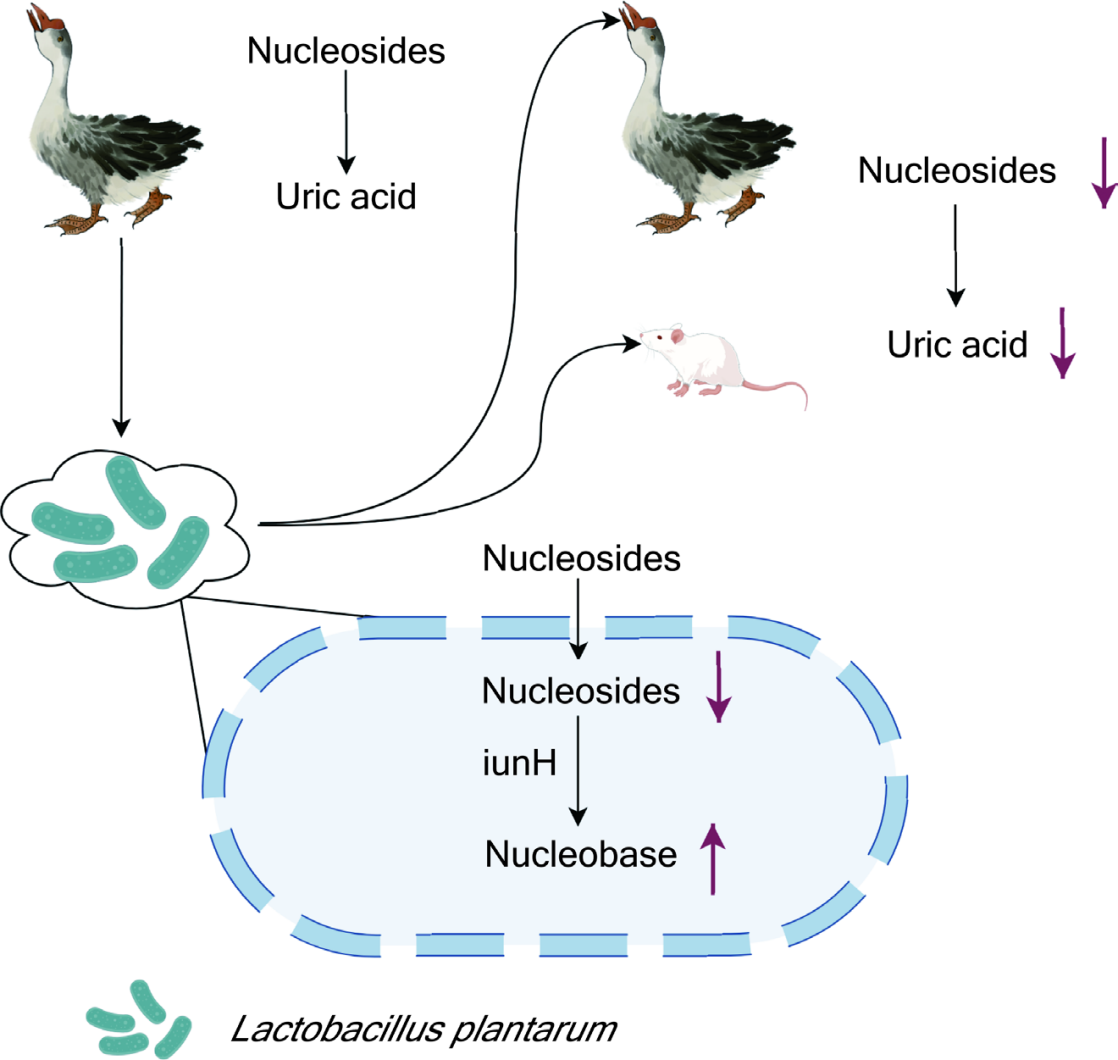
***Lactobacillus plantarum*** **reduces uric acid synthesis through the hydrolysis of nucleosides.** A strain of the bacterium *L. plantarum* was isolated from the large intestine of geese with hyperuricemia, a condition caused by the presence of abnormally high levels of uric acid in the blood (top left). In vitro experiments showed that the presence of the bacteria led to an increase in the degradation of nucleosides that are precursors of uric acid. Administering the bacteria to healthy geese and mice (top right) also led to a reduction in the levels of uric acid in the blood. Other experiments showed that *L. plantarum* absorbed the nucleosides, and that an enzyme called iunH broke down the nucleosides to produce nucleobases (bottom).

Figure created with figdraw.com.
